# Ursodeoxycholic acid inhibits uptake and vasoconstrictor effects of taurocholate in human placenta

**DOI:** 10.1096/fj.201900015RR

**Published:** 2019-03-28

**Authors:** Emma M. Lofthouse, Christopher Torrens, Antigoni Manousopoulou, Monica Nahar, Jane K. Cleal, Ita M. O’Kelly, Bram G. Sengers, Spiros D. Garbis, Rohan M. Lewis

**Affiliations:** * Faculty of Medicine, University of Southampton, Southampton, United Kingdom;; † Institute for Life Sciences, University of Southampton, Southampton, United Kingdom;; ‡ Faculty of Engineering, University of Southampton, Southampton, United Kingdom; and; § Division of Biology and Biological Engineering, Proteome Exploration Laboratory, Beckman Institute, California Institute of Technology, Pasadena, California, USA

**Keywords:** OATP4A1, membrane transport, vasoconstriction

## Abstract

Intrahepatic cholestasis of pregnancy (ICP) causes increased transfer of maternal bile acids to the fetus and an increased incidence of sudden fetal death. Treatment includes ursodeoxycholic acid (UDCA), but it is not clear if UDCA protects the fetus. This study explores the placental transport of the bile acid taurocholate (TC) by the organic anion–transporting polypeptide, (OATP)4A1, its effects on the placental proteome and vascular function, and how these are modified by UDCA. Various methodological approaches including placental villous fragments and *Xenopus laevis* oocytes were used to investigate UDCA transport. Placental perfusions and myography investigated the effect of TC on vasculature. The effects of acute TC exposure on placental tissue were investigated using quantitative proteomics. UDCA inhibited OATP4A1 activity in placental villous fragments and oocytes. TC induced vasoconstriction in placental and rat vasculature, which was attenuated by UDCA. Quantitative proteomic analysis of villous fragments showed direct effects of TC on multiple placental pathways, including oxidative stress and autophagy. The effects of TC on the placental proteome and vasculature demonstrate how bile acids may cause fetal distress in ICP. UDCA inhibition of OATP4A1 suggests it will protect the mother and fetus against the vascular effects of TC by inhibiting its cellular uptake. UDCA may protect the fetus in ICP by inhibiting OATP4A1-mediated bile acid transfer and TC-induced placental vasoconstriction. Understanding the physiologic mechanisms of UDCA may allow better therapeutic interventions to be designed specifically for the fetus in the future.—Lofthouse, E. M., Torrens, C., Manousopoulou, A., Nahar, M., Cleal, J. K., O’Kelly, I. M., Sengers, B. G., Garbis, S. D., Lewis, R. M. Ursodeoxycholic acid inhibits uptake and vasoconstrictor effects of taurocholate in human placenta.

Intrahepatic cholestasis of pregnancy (ICP) is a disease characterized by elevated maternal and fetal plasma bile acids, which may have severe short-term and long-term consequences for the fetus (1, 2). Although maternal prognosis is good, ICP poses significant risks, including fetal distress, preterm delivery, and, in severe cases, spontaneous fetal death (3).

The 2 classes of primary bile acids are cholic acid and chenodeoxycholic acid, which are synthesized from cholesterol in the liver and converted to secondary bile acids (taurocholic acid, glychocholic acid, glycochenodeoxycholic acid, and taurochenodeoxycholic acid) by conjugation or enzymatic modification, which occurs in the liver, or by intestinal bacteria. Within these classes of primary and secondary bile acids, multiple subtypes are present, representing different chemical modifications (4, 5). Bile acids are secreted into the duodenum and, after facilitating lipid absorption, are reabsorbed from the terminal ilium and recycled to the liver.

From early on in gestation, the fetal liver synthesizes bile acids, which are removed from the fetal circulation by placental transfer to the maternal circulation. However, in ICP, high maternal levels of bile acids result in reversed transfer and may also competitively inhibit physiologic transfer of fetal bile acids to the mother (5). Consequently, these 2 factors result in a significant accumulation of maternal bile acids in the fetal circulation. It is not clear how increased bile acid concentrations have adverse effects on the fetus, but there is evidence that they increase smooth muscle contractility in the placental vasculature and reduce synchronicity in fetal cardiomyocytes (6–8). Elevated bile acid concentrations could adversely affect placental function, leading to secondary effects on the fetus. For example, changes in placental membrane transporter expression have been reported, with the organic anion–transporting polypeptide (OATP)4A1 and OATP2B1 mRNAs being up-regulated and down-regulated, respectively, in ICP placentas (9, 10), which may affect placental function.

To cross the placenta, bile acids need to be transported across both the maternal-facing microvillous membrane and the fetal-facing basal membrane of the placental syncytiotrophoblast as well as the fetal capillary endothelium. It is unclear whether the placenta metabolizes maternal or fetal bile acids. A deeper understanding of how bile acids are transported across the polarized syncytiotrophoblast can provide insight as to how their transfer to the fetus can be inhibited.

A variety of transporters are expressed in the placenta that could facilitate bile acid transfer in both the maternal and fetal directions. These include the OATPs such as OATP4A1, which has been reported to be up-regulated in cholestasis placentas (11), OATP2B1, and the breast cancer resistance protein efflux pump (12). In the liver, the bile salt export pump and the sodium taurocholate (TC) cotransporting polypeptide SLC10A1 are the major bile acid transporters, but their expression in the placenta is negligible (13).

The treatment of ICP currently involves administering a naturally occurring bile acid, ursodeoxycholic acid (UDCA), to alleviate maternal symptoms by altering the composition of the bile acid pool (14). The mechanisms behind this mode of action are unclear, although it is reported that UDCA reduces apoptosis, villitis of unknown etiology, and bile acid–induced oxidative stress and inflammatory effects on placental trophoblasts (14–16). UDCA is reported to be safe for the fetus, but it is unknown what effects UDCA has on placental function (17, 18). Although UDCA is prescribed in clinical practice, there is not a consensus as to whether UDCA is beneficial (19). There is a need for a broader physiological understanding of how UDCA may affect the placenta because this could change placental function and alter fetal development (19).

The primary purpose of this study was to investigate the transport of the bile acid TC across the human placenta and the effects of UDCA on bile acid transport and placental function. Because OATP4A1 is reportedly up-regulated in ICP, a combination of placental villous fragments and OATP4A1-expressing *Xenopus laevis* oocytes were used to investigate the uptake of TC and UDCA across the microvillous membrane of the placental syncytiotrophoblast, whereas human placental perfusions and myography were used to investigate the mechanisms underlying bile acid–mediated vasoconstriction.

## MATERIALS AND METHODS

### Study approval

Placentas were collected with written informed consent from women delivering at the Princess Anne Hospital in Southampton with approval from the Southampton and Southwest Hampshire Local Ethics Committee (11/SC/0529).

All animal procedures were performed in accordance with the regulations of the British Home Office Animals (Scientific Procedures) Act 1986 and approved by the local Ethical Review Committee. For myography studies, male Wistar rats (300–400 g) from Charles River Laboratories (Wilmington, MA, USA) were used.

### cRNA synthesis for microinjection into oocytes

The plasmid containing the cDNA of human OATP4A1 [Solute carrier organic anion transporter family member 4A1 (SLCO4A1)] was obtained from Origene (Rockville, MD, USA) and was linearized using the *Streptomyces achromogenes* *Sac*I restriction enzyme (Promega; Madison, WI, USA). Because the plasmid contained 2 bacteriophage T7 (T7) promoter sites, PCR was used to amplify the region containing only the first T7 promoter and the SLCO4A1 gene before purification to prevent synthesis of cRNA from the noncoding T7 site (forward primer: 5′-GCACCAAAATCAACGGGACT-3′, reverse primer: 5′-CAACGGCACTGTTCTGTCAT-3′). cRNA was synthesized from the coding T7 promoter using the Ambion mMachine mMessage Kit (Thermo Fisher Scientific, Waltham, MA, USA) according to the manufacturer’s instructions.

### *Xenopus* oocyte transstimulation studies

*Xenopus laevis* oocytes were used as an expression system for the characterization of OATP4A1.

*Xenopus laevis* oocytes were obtained from the European *Xenopus* Resource Centre (Portsmouth, United Kingdom). Oocytes were treated with collagenase (2 mg/ml) in buffer OR2 (2.5 m KCl, 82.5 mM NaCl, 1 mM CaCl_2_, 1 mM Na_2_HPO_4_, 1 mM MgCl_2_, and 5 mM HEPES) for 1 h at room temperature. Oocytes were then incubated in ND91 buffer (2 mM KCl, 91 mM NaCl, 1.8 mM CaCl_2_, 1 mM MgCl_2_, 5 mM HEPES, 1% penicillin-streptomycin, and 0.1% gentamycin sulfate) overnight at 18°C. Stage V oocytes were injected with 20 ng of OATP4A1 cRNA dissolved in 38 nl of water. The water-injected control oocytes were injected with an equivalent volume of water.

#### ^3^H-estrone sulfate uptake studies

OATP4A1 transports a wide variety of steroid hormones and drugs in addition to bile acids. Previously, *X. laevis* oocytes were found to endogenously express a carrier system that mediated TC efflux (20). As a result, the ^3^H-estrone sulfate (^3^H-ES) was used as a model substrate for inhibiting OATP4A1 transporter activity. Two to three days after injection of OATP4A1 cRNA, individual oocytes were transferred to single wells of a 96-well plate and incubated with ^3^H-ES (11 μM) in ND91 buffer for 2.5–30 min with or without the presence of 2.5 mM glutamate or ES to inhibit uptake. Uptake was then stopped by washing the oocytes 3 times in cold ND91 buffer. Each oocyte was homogenized in 5% SDS, and levels of tracer were analyzed by liquid scintillation counting.

#### ^3^H-ES efflux studies

To determine whether OATP4A1 mediates UDCA transport, individual OATP4A1 cRNA and water-injected oocytes were incubated with ^3^H-ES (6 nM) for 1 h in sodium-free ND91. Each oocyte was washed 3 times in warm sodium-free ND91 before 100 μl of buffer alone, 500 μM ES, 500 μM UDCA, or 500 μM glycine was added for 5 min to stimulate efflux of ^3^H-ES.

Efflux was stopped after 5 min, and the efflux buffer of 5 individual oocytes was combined (500 μl total) and analyzed by liquid scintillation counting (5 oocytes/condition, 3 replicates). Each independent experiment used oocytes from a different frog and was conducted on a different week.

### Placental villous fragments

#### Radiolabeled uptake time-course experiments

Villous fragments (10 mg) were dissected from human term placentas and cultured for 0, 1, 2.5, 5, 7.5, 10, 30, or 60 min in Tyrode’s buffer [135 mM NaCl, 5 mM KCl, 1.8 mM CaCl_2_, 1 mM MgCl_2_6.H_2_O, 10 mM HEPES, and 5.6 mM glucose (pH 7.4)] containing 5 nM ^3^H-ES (9.25 MBq, NET203250UC; PerkinElmer, Waltham, MA, USA) (*n* = 3 placentas, duplicate conditions, 3 fragments per replicate) or 16 nM ^3^H-taurocholic acid (^3^H-TC) (9.25 MBq, NET322250UC; PerkinElmer). Uptake was stopped by washing the fragments in cold Tyrode’s buffer before homogenizing the fragments in NaOH (50 mM). Uptake of ^3^H-ES and ^3^H-TC was determined by liquid scintillation counting (PerkinElmer).

#### Uptake inhibition experiments

Villous fragments (10 mg) were dissected from human term placentas and cultured for 2.5 min or 1 min (as determined by time-course experiments) in Tyrode’s buffer containing 26 nM ^3^H-ES or 106 nM ^3^H-TC, respectively (*n* = 5 placentas), with or without 100 μM–1 mM ES (*n* = 5 placentas), glycine (*n* = 5 placentas), TC (*n* = 5 placentas), or 10 μM–1 mM UDCA (*n* > 3 placentas). To ensure complete inhibition of OATP4A1-mediated transport, the concentrations of these substrates were used above physiological levels. Conditions were done in triplicate with 3 fragments per replicate. Uptake of ^3^H-ES or ^3^H-TC was stopped by washing the fragments in cold Tyrode’s buffer before homogenizing the fragments in NaOH (50 mM). Uptake of the tracer was determined by liquid scintillation counting (PerkinElmer).

#### Proteomics experiments

Villous fragments (10 mg) were dissected from human term placentas and cultured for 8 h in Tyrode’s buffer alone or 100 μM TC in Tyrode’s buffer (*n* = 3 placentas, 3 replicate conditions, 3 fragments/replicate).

### Vascular function

Placentas were perfused using the methodology of Schneider *et al.* (21), as adapted in our laboratory (22). Placentas were perfused with Earle’s Bicarbonate Buffer (5 mM glucose, 1.8 mM CaCl_2_, 0.4 mM MgSO_4_, 116.4 mM NaCl, 5.4 mM KCl, 26.2 mM NaHCO_3_, and 0.9 mM NaH_2_PO_4_) and gassed with 5% CO_2_ and 95% O_2_ *via* the fetal catheter going into the chorionic plate fetal artery at 6 ml/min and *via* 5 maternal catheters at 14 ml/min using a roller pump. At 20-min intervals, boli of 1 μM TC (estimated maximal concentration in tissue after dilution 380 μM) and 20 nM angiotensin were perfused into the fetal circulation (*n* = 8). Throughout the experiment, fetal and maternal placental circulation pressures were recorded using Chart v.4.2 (ADInstruments, Oxford, United Kingdom).

### Myography

#### Human placental arteries

Arteries from the human term placental chorionic plate were mounted on a wire myograph (Danish Myo Technology, Aarhus, Denmark) in physiologic salt solution (PSS) of the following composition: NaCl, 119; KCl, 4.7; CaCl_2_, 2.5; MgSO_4_, 1.17; NaHCO_3_, 25; KH_2_PO_4_, 1.18; EDTA, 0.026; and d-glucose, 5.5 mM. Segments were bathed in PSS, heated to 37°C, and continuously gassed with 95% O_2_ and 5% CO_2_. Smooth muscle integrity was assessed by a single wash with 125 mM PSS solution with an equimolar substitution of KCl for NaCl. Following equilibration, cumulative concentration response curves (CRCs) were constructed to the bile acid TC (1 nM–100 μM, *n* = 5 placentas) in the absence or presence of the organic anion transporter (OAT) inhibitor ES (100 μM, *n* = 3 placentas) and the Ca^2+^ channel blocker nifedipine (1 μM, *n* = 2 placentas). Responses to the thromboxane mimetic U46619 (1 μM, *n* = 3 placentas) were also performed.

#### Rat thoracic aorta

Male Wistar rats (350–450 g; Charles River Laboratories) were humanely killed by CO_2_ inhalation and cervical dislocation. The thoracic aorta was dissected and cut into 2-mm segments and mounted on a myograph as previously described. Thoracic aorta segments were placed under a resting tension of 1 g, and smooth muscle integrity was assessed by a single wash with 125 mM PSS solution with an equimolar substitution of KCl for NaCl. Following equilibration, cumulative CRCs were constructed to the α_1_-adrenoceptor agonist phenylephrine (1 nM–100 μM, *n* = 13). Cumulative CRCs were then constructed to the bile acid TC (1 nM–100 μM, *n* = 13) alone or in the presence of nifedipine (1 μM, *n* = 3) and the OAT inhibitors UDCA (100 μM, *n* = 5), ES (100 μM, *n* = 4), and bromosulphothalein (100 μM, *n* = 7).

### Quantitative proteomics sample processing

Placental villous fragments were snap frozen at −80°C. These were dissolved in 0.5 M triethylammonium bicarbonate and 0.05% SDS and subjected to pulsed probe sonication (Misonix, Farmingdale, NY, USA). Lysates were centrifuged (16,000 *g*, 10 min, 4°C) and supernatants were measured for protein content using infrared spectroscopy (MilliporeSigma, Burlington, MA, USA). Lysates were then reduced, alkylated, and subjected to trypsin proteolysis. Peptides were labeled using the eight-plex iTraq reagent kit (MilliporeSigma; 113 = control 1, 114 = TC 1, 115 = control 2, 116 = TC 2, 117 = control 3, 118 = TC 3, 119 = control 4, and 121 = TC 4) and analyzed using 2-dimensional liquid chromatography (offline alkaline C_4_ reverse phase and online acidic C_18_ reverse phase) and tandem mass spectrometry as previously reported by the authors (23, 24).

### Database searching

Unprocessed raw files were submitted to Proteome Discoverer 1.4 (Thermo Fisher Scientific) for target decoy searching against the UniProt Knowledgebase *Homo sapiens* database (*https://www.uniprot.org/proteomes/UP000005640*) composed of 20,159 entries (release date, January 2015), allowing for up to 2 missed cleavages, a precursor mass tolerance of 10 ppm, a minimum peptide length of 6, and a maximum of 2 variable (1 equal) modifications of 8-plex iTraq (Y), oxidation (M), deamidation (N, Q), or phosphorylation (S, T, and Y). Methylthio (C) and iTraq (K, Y, and N terminus) were set as fixed modifications. False discovery rate at the peptide level was set at <0.05. Percent coisolation excluding peptides from quantitation was set at 50. Reporter-ion ratios from unique peptides only were taken into consideration for the quantitation of the respective protein. A 1-sample Student’s *t* test using the normalized iTraq ratios of each TC-treated placenta compared with its respective control was performed. Significance was set at *P* ≤ 0.05. In adherence to the Paris Publication Guidelines for the analysis and documentation of peptide and protein identifications (*https://doi.org/10.1074/mcp.T400006-MCP200*) only proteins identified with at least 2 unique peptides were further subjected to bioinformatics. All mass spectrometry data have been deposited to the ProteomeXchange Consortium *via* the Proteomics Identifications Archive with the data set identifier PXD009825.

### Bioinformatics analysis

The Database for Annotation, Visualization and Integrated Discovery (DAVID; *https://david.ncifcrf.gov/*) was applied to differentially expressed proteins in order to identify overrepresented gene ontology (GO) terms. A Fisher exact–corrected value of *P* ≤ 0.05 was considered significant.

### Statistics

^3^H-ES and ^3^H-TC uptake in OATP4A1 oocytes and placental villous fragments was analyzed by a 1-way ANOVA with a Dunnett’s *post hoc* test, in which ^3^H-ES or ^3^H-TC uptake in the presence of OATP4A1 substrates was compared with the tracer alone.

^3^H-ES efflux in OATP4A1 oocytes was analyzed by a 1-way ANOVA with a Dunnett’s *post hoc* test, in which ^3^H-ES efflux stimulated by OATP4A1 substrates was compared with ^3^H-ES efflux in response to buffer alone. Significance was assumed at a value of *P* < 0.05, and oocyte data were adjusted for water-injected responses and presented as means ± sem.

Placental perfusion peak force data were adjusted for baseline pressure and analyzed using an unpaired Student’s *t* test in which data were compared with a hypothetical value of 0. Significance was assumed at a value of *P* < 0.05. Data are presented as means ± sem.

Responses in rat aortas and chorionic plate vessels were measured as the percentage of maximum response in the presence or absence of inhibitor. Data are presented as means ± sem, and significance was assumed at a value of *P* < 0.05.

## RESULTS

### Human tissue data

Placentas used in this study were obtained from normal-term deliveries, of which 70% were caesareans and with an average parity of 1.5.

### OATP4A1 activity is inhibited by UDCA

**Figure 1:**
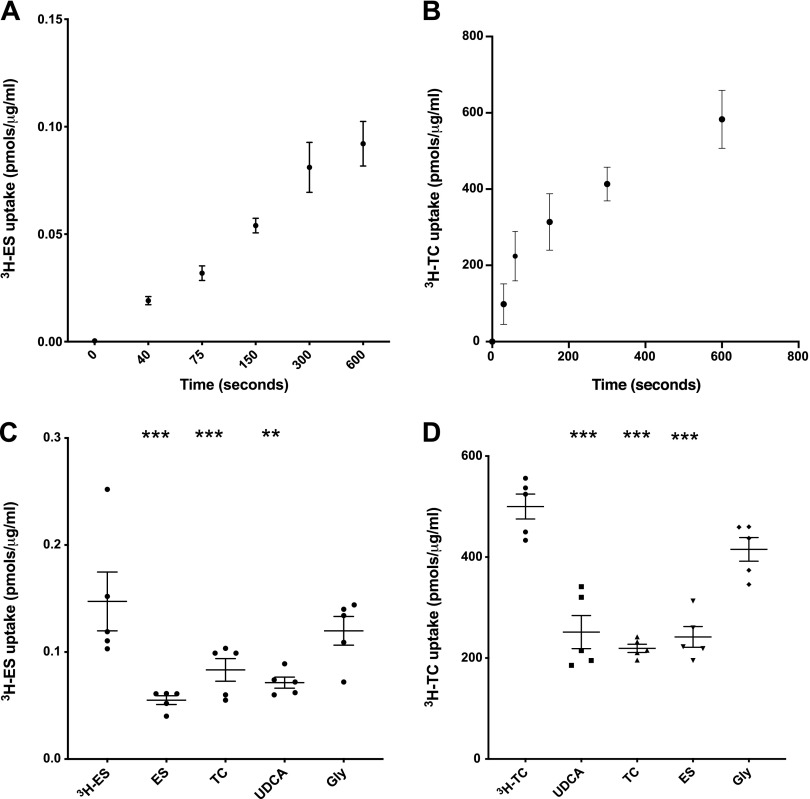
The uptake of OATP substrates, ES and TC, are inhibited by UDCA in placental villous fragments. *A*) ^3^H-ES uptake by villous fragments was shown to be linear up to 5 min (*n* = 2 placentas). *B*) ^3^H-TC uptake by villous fragments was shown to be linear up to 2.5 min (*n* = 3 placentas). *C*) ^3^H-ES uptake was inhibited by 1 mM ES (***P < 0.001, *n* = 5), 1 mM TC (***P < 0.001, *n* = 5), and 1 mM UDCA (**P < 0.01, *n* = 5) but not by the negative control glycine (*n* = 5). *D*) ^3^H-TC uptake was inhibited by 100 μM UDCA (***P < 0.01, *n* = 5), 100 μM TC (**P < 0.01, *n* = 5), and 100 μM ES (***P < 0.01, *n* = 5), but not by the negative control glycine (*n* = 5). Data are presented as means and sem and were analyzed by a 1-way ANOVA with Dunnett’s *post hoc* (compared with ^3^H-ES and ^3^H-TC alone).

**Figure 2:**
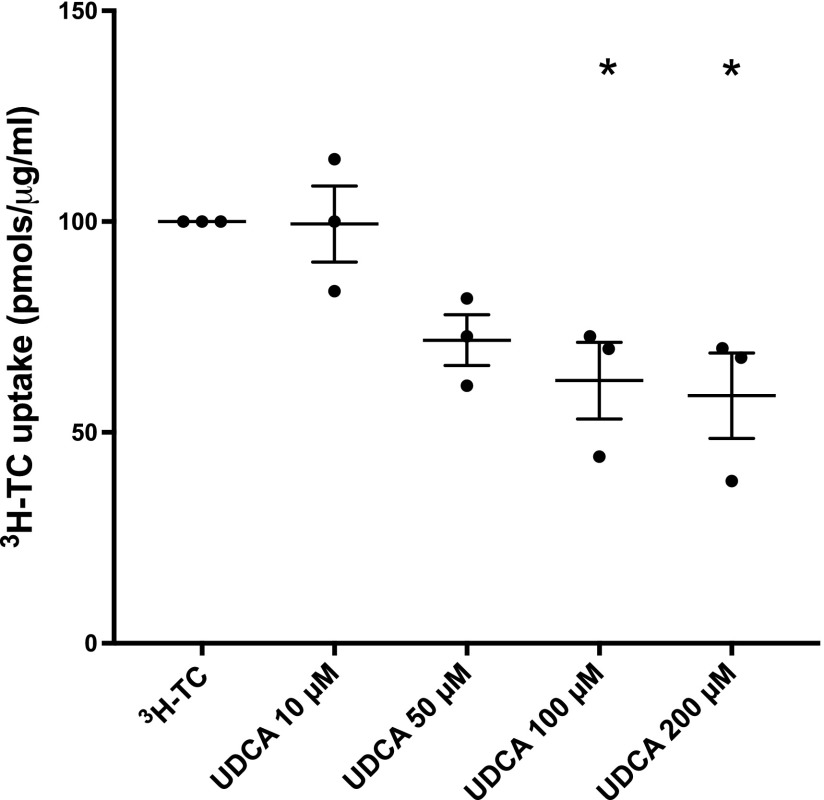
UDCA inhibits TC uptake in placental villous fragments in a dose-dependent manner. Compared with ^3^H-TC alone, 100–200 μM UDCA inhibited TC uptake (*P < 0.05, *n* = 3 placentas, triplicate conditions, 3 fragments/replicate). Data are presented as means and sem and were analyzed by a 1-way ANOVA with Dunnett’s *post hoc* (compared with ^3^H-TC alone).

Placental villous fragment uptake experiments were carried out to investigate OATP-mediated ES and TC uptake. In placental villous fragments, uptake of the OATP4A1 substrate ^3^H-ES was linear up to 10 min (*n* = 2 placentas, **Fig. 1*A***), whereas uptake of ^3^H-TC was linear up to 2 min (*n* = 3 placentas, Fig. 1*B*). Subsequent uptake experiments in villous fragments were carried out at the 2.5 min time point for ^3^H-ES and 1 min for ^3^H-TC. ^3^H-ES uptake was inhibited by 1 mM ES (*P* < 0.001, *n* = 5 placentas), 1 mM TC (*P* < 0.001, *n* = 5 placentas), and 1 mM UDCA (*P* < 0.006, *n* = 5 placentas; Fig. 1*C*). ^3^H-TC uptake was inhibited by 100 μM TC (*P* < 0.001, *n* = 5 placentas), 100 μM ES (*P* < 0.001, *n* = 5 placentas), and 100 μM UDCA (*P* < 0.001, *n* = 5 placentas; Fig. 1*D*). ^3^H-TC uptake was also inhibited by 200 μM UDCA (*P* < 0.05, *n* = 3 placentas) but not by 10 or 50 μM UDCA (**Fig. 2**).

**Figure 3:**
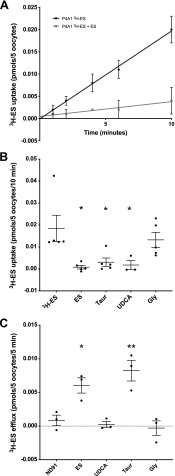
UDCA inhibits OATP4A1 but does not cause transstimulation, suggesting it is not transported. *A*) ^3^H-ES uptake into oocytes by OATP4A1 was shown to be linear up to 10 min, and this was inhibited by cold 2.5 mM ES (*n* = 5 ovaries, 5 oocytes per condition). *B*) ^3^H-ES uptake (under sodium-free conditions) in OATP4A1-injected oocytes was inhibited by 500 μM ES, TC, and UDCA (*P < 0.05, *n* = 5 ovaries, 5 oocytes/condition), but not by negative control glycine. Data are presented as means and sem and are analyzed by a 1-way ANOVA with Dunnett’s *post hoc* (compared with ^3^H-ES alone). *C*) ^3^H-ES efflux in OATP4A1-injected oocytes is transstimulated by ES (*P < 0.05, *n* = 3) and TC (**P < 0.01), but not by UDCA or negative control glycine. This suggests that UDCA is not a substrate of OATP4A1. Data are adjusted for background water-injected responses and presented as means and sem. Data were analyzed by a 1-way ANOVA with Dunnett’s *post hoc* (compared with buffer alone); *n* > 3 individual ovaries, 5 oocytes per condition for uptake, 15 oocytes/condition for efflux.

*Xenopus* oocytes were injected with water alone or SLCO4A1 cRNA in order to express the OATP4A1 transporter so transport function could be investigated. In *Xenopus* oocytes expressing the OATP4A1 protein, ^3^H-ES uptake was linear for 10 min and was inhibited by cold 2.5 mM ES (**Fig. 3*A***). Subsequent uptake experiments were performed at 10 min. ^3^H-ES uptake (under sodium-free conditions) in oocytes injected with OATP4A1 cDNA was inhibited by 500 μM ES, TC, and UDCA (*P* < 0.05) but not by the negative control glycine (Fig. 3*B*).

### UDCA is not transported by OATP4A1

In OATP4A1-expressing *Xenopus* oocytes, 500 μM of OATP4A1 substrate ES (*P* = 0.02, *n* = 3 ovaries, triplicate conditions) and 500 μM TC (*P* = 0.002, *n* = 3 ovaries) transstimulated efflux of ^3^H-ES (Fig. 3*C*). Efflux was not stimulated by buffer alone, 500 μM UDCA (*n* = 3 ovaries), or the negative control glycine (*n* = 3 ovaries).

### TC induces vasoconstriction in human placental vasculature

**Figure 4:**
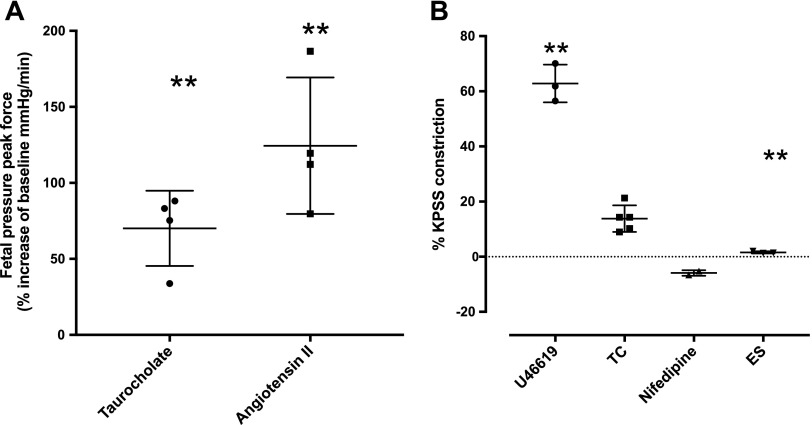
TC induces vasoconstriction in human placental vasculature. *A*) In placental perfusions, infusion of a 1 μM TC bolus into the fetal circulation (**P < 0.01, *n* = 4 perfusions) and subsequently 20 nM angiotensin II (**P < 0.011, *n* = 4 perfusions) significantly increased fetoplacental pressure from baseline. Data are presented as means and sem and were analyzed using an unpaired Student’s *t* test in which data were compared with a hypothetical value of 0. *B*) In chorionic plate arteries from human term placenta, TC (*n* = 5) produced a modest constriction that was significantly less potent than the thromboxane mimetic U46619 (1 μM) (*n* = 3, **P < 0.01). Although this was a modest response, it was abolished by both nifedipine (1 μM, = 2) and ES (100 μM, *n* = 3, **P < 0.01).

Following an infusion of a 1-μM TC bolus into the fetal circulation of an *ex vivo–*perfused human placental cotyledon (final maximal tissue concentration, 380 μM), pressure in the fetoplacental circulation increased 71% from baseline (*P* = 0.01, *n* = 4 placentas, **Fig. 4*A***). A bolus of the vasoconstrictor angiotensin II (20 nM) was injected into the fetoplacental circulation as a positive control and increased fetoplacental pressure by 125% from baseline (*P* = 0.01, *n* = 4 placentas). Placentas that did not show vasoconstriction in response to angiotensin II were removed from the analysis.

### Myography in human chorionic plate vessels and in rat aorta

To better characterize vascular responsiveness to TC, myography was performed on isolated vessels. In chorionic plate arteries from human term placenta, TC (*n* = 5) produced a modest constriction (Fig. 4*B*) that was significantly less potent than the thromboxane mimetic U46619 (1 μM, *n* = 3). Although this was a modest response, it was abolished by both the calcium channel blocker nifedipine (1 μM, *n* = 2) and OAT inhibitor ES (100 μM, *n* = 3, *P* < 0.01; Fig. 4*B*).

**Figure 5:**
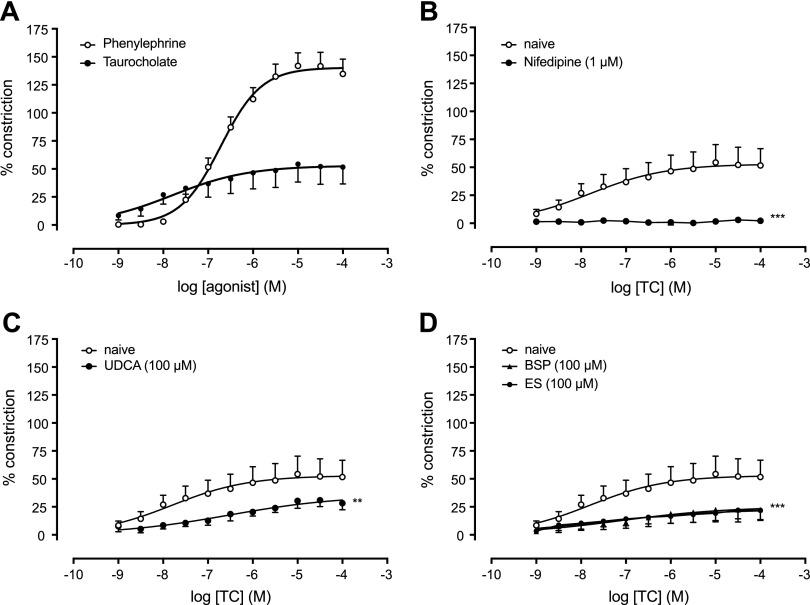
TC-induced concentration-dependent vasoconstriction in rat aortas is inhibited by OATP substrates, UDCA, and the calcium channel blocker nifedipine. *A*) Vasoconstriction is induced by phenylephrine and TC (*n* = 13). *B*) TC responses were abolished in the presence of 1 μM nifedipine, a calcium channel blocker (*n* = 3, ***P < 0.005). *C*) TC constriction is abolished in the presence of UDCA (*n* = 5, **P < 0.01). *D*) TC constriction is abolished in the presence of OAT inhibitor BSP (*n* = 7, ***P < 0.005) and ES (*n* = 4, ***P < 0.005).

Because responses to TC in human placental chorionic plate arteries were modest, rat aorta was used to allow a fuller analysis of TC responsiveness. In rat aortas, TC (*n* = 13) produced a concentration-dependent vasoconstriction (**Fig. 5*A***) that was significantly less potent than phenylephrine (*n* = 13; Fig. 5*A*) and was abolished in the presence of nifedipine (1 μM, *n* = 3, *P* = 0.001; Fig. 5*B*). Responses to TC were also abolished in the presence of the OAT inhibitors bromosulphothalein (100 μM, *n* = 7, *P* = 0.0003), ES (100 μM, *n* = 4, *P* = 0.005), and UDCA (100 μM, *n* = 5, *P* = 0.006; Fig. 5*C, D*).

### Proteomics

**Figure 6:**
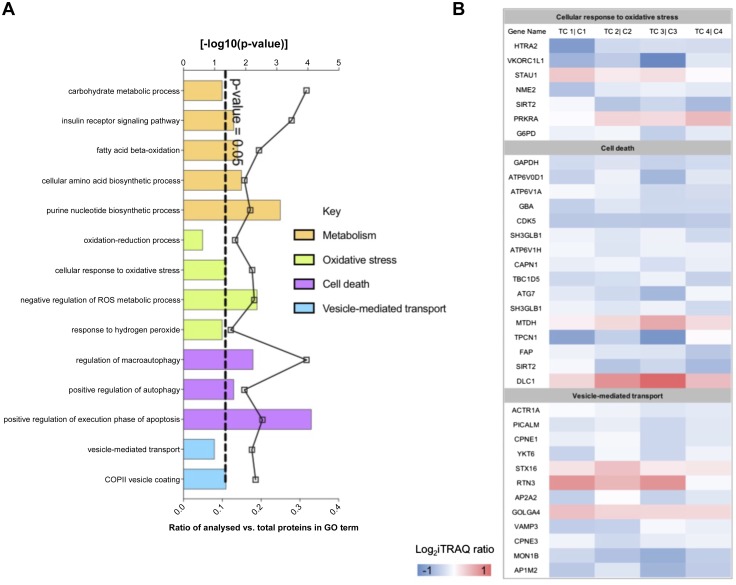
*A*) GO analysis of the differentially expressed proteins following 8 h exposure of placental villous fragments to TC *vs.* controls showed significant enrichment for GO terms related to metabolism, cell death, oxidative stress, and vesicle-mediated transport. The dotted line indicates a value of *P* = 0.05. *B*) Differentially expressed proteins that map to significant pathways (cellular response to oxidative stress, cell death, and vesicle-mediated transport) presented in heatmap format representing the ratio of expression in treated *vs.* control samples for each placenta.

The quantitative proteomic analysis of term placental villous fragments exposed for 8 h to TC (100 μM, *n* = 4 placentas) compared with patient-matched controls profiled 7908 unique protein groups (peptide level FDR, *P* < 0.05). Of these proteins, 615 were differentially expressed following 8 h exposure to TC compared with control (Student’s *t* test, *P* < 0.05). GO analysis of the differentially expressed proteins following 8 h exposure to TC *vs.* controls showed significant enrichment for GO terms related to metabolism, cell death, oxidative stress, and vesicle-mediated transport (DAVID, Fisher exact–corrected value of *P* ≤ 0.05; **Fig. 6*A***). The differentially expressed proteins that map to cellular response to oxidative stress, cell death, and vesicle-mediated transport are presented in heatmap format in Fig. 6*B*.

## DISCUSSION

This study provides novel insight into TC uptake by the human placenta as well as its effects on vascular reactivity and protein-level perturbation of biologic pathways and networks. We show that UDCA is a competitive inhibitor of OATP4A1-mediated TC uptake, and as such, UDCA can inhibit the vascular effects of TC. The effects of TC on the placenta and its interaction with UDCA are directly relevant to our understanding of the effects of ICP and improving treatment for this condition, which is associated with increased fetal death incidence.

We demonstrate for the first time that TC is a vasoactive bile acid in the human placenta, capable of raising perfusion pressure in isolated perfused placental cotyledons and constricting isolated chorionic plate arteries. The effect of TC appeared stronger in the perfused placentas compared with the chorionic plate arteries. This may indicate that the site of TC action lies deeper within the placenta or that the increase in resistance observed in the perfused placenta represents the sum of a smaller effect across the placental vasculature. In either case, TC-induced vasoconstriction at the level seen in the perfused placenta would increase work required by the fetal heart in order to maintain adequate placental perfusion. Because TC may also affect fetal cardiomyocytes, these attributes may collectively contribute to the overall increased rate of fetal death seen in ICP (25, 26).

**Figure 7:**
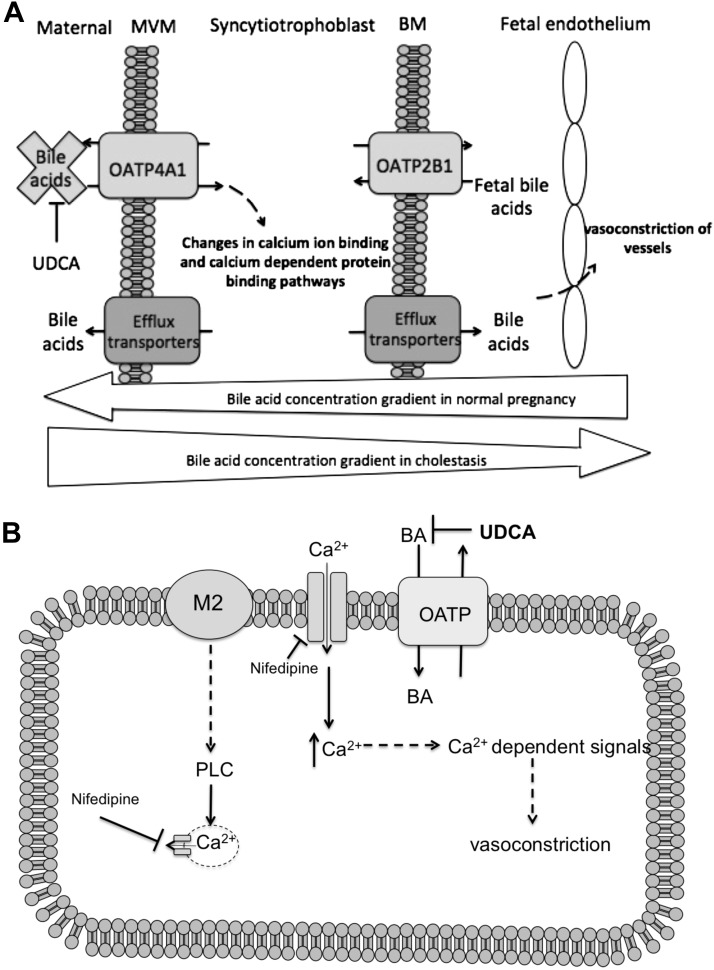
*A*) In cholestasis, high maternal bile acid concentrations lead to reversal of the normal direction of bile acid transfer being reversed. Placental OATP4A1 activity inhibition *via* UDCA prevents toxic bile acid uptake across the microvillous membrane of the placental syncytiotrophoblast. *B*) We propose that vasoconstriction of placental smooth muscle results from altered calcium signaling induced by a rise in intracellular calcium from M2 receptors and l-type calcium channels.

We subsequently characterized the mechanisms involved in mediating TC-induced vasoconstriction in rat aortas. These data show that TC-induced vasoconstriction is mediated, in part, *via* muscarinic cholinergic receptors and by OATP transporters. Because constriction was inhibited by known OATP inhibitors and UDCA, this indicates that TC is transported into the cell to induce its systemic effect (**Fig. 7**). Within the cell, we hypothesize that TC or a metabolite can induce calcium entry, leading to constriction of the vascular smooth muscle.

UDCA is used as a maternal therapy in ICP, but little is known about its transport mechanism across the placenta. This study sought to determine whether UDCA is transported by OATP4A1, which is known to utilize the placental glutamate gradient to transport TC (11, 27). UDCA was found to be an inhibitor of OATP4A1 but did not transstimulate its activity, so it does not appear to be a substrate, as has been suggested by previous studies (28). Because OATP4A1 does not appear to transport UDCA, this raises the possibility that an unknown transporter or mechanism is implicated in the transfer of UDCA to the fetus.

The fetal protective effects of UDCA may be associated with its inhibition of OATP4A1, which in turn prevents the uptake of bile acids into the placenta. Consequently, apoptosis-related pathways against the fetoplacental vasculature and on the fetus itself are mitigated (29). There is some debate about the use of UDCA in ICP because the mechanisms of action are unclear and benefits for the fetus are unknown (19). This study suggests that the administration of UDCA is likely to have direct and indirect benefits for fetal health by maintaining placental health and preventing bile acid transport, respectively.

The agnostic proteomic analysis provided novel protein-level evidence that short-term exposure of placental villi to TC significantly affected multiple pathways. These pathways included those related to metabolism, oxidative stress, and autophagy, consistent with previous studies on ICP placentas (30). This study demonstrates that these changes in the proteome are direct effects of TC and not secondary to the effects of ICP on the mother. Changes in metabolic, oxidative stress, and autophagy pathways could alter placental function in ways that are detrimental to the fetus, especially with chronic exposure. Changes in the expression of proteins associated with calcium physiology (*e.g.*, the calcium-regulating binding protein calreticulin) may be implicated in the vasoactive effects observed, but specific studies on smooth muscle would be required to determine this.

In cholestasis, stillborn fetuses and their placentas are reportedly hypoxic with high levels of oxidative stress associated with prolonged constriction of placental chorionic veins (31). In the present study, oxidative stress pathways were also significantly altered in response to bile acid exposure because ferredoxin 1, a protein involved in bile acid metabolism and transferring electrons into the cytochrome p450 system, was up-regulated. Vascular cell adhesion protein 1, an inflammatory gene, was also one of the most up-regulated proteins, whereas the TP53-inducible glycolysis and apoptosis regulator, a gene that regulates glucose breakdown and promotes DNA repair, was one of the most down-regulated proteins, which may affect nutrient transfer as a result (32).

This investigation provides mechanistic insight into the transport of bile acids across the placenta and describes the vascular effects of bile acids as well as effects on placental function, which may have implications for ICP. The myography and perfusion work in this study demonstrate that bile acids mediate vasoconstriction that may directly impact fetal health. Previously, bile acids have been demonstrated to cause a loss of synchronicity of contraction in rat fetal cardiomyocytes (26, 29). Moreover, conditions such as absent or reversed end-diastolic flow and hypoxia are known to cause fetal distress and reduced fetal growth and are similarly associated with vasoconstriction and poor placental perfusion, as we report here (33, 34).

This study indicates that the vascular effects of TC require it to be transported into the cell. Future research into limiting the effects of cholestasis on the mother or the fetus may focus on inhibiting the uptake of bile acids into cells or on understanding how bile acids have their effects within the cell. The use of UDCA or a higher-affinity analog would have promise because it would block bile acid transfer by OATP4A1 but does not seem to be transported by it, so it would not itself affect the intracellular milieu. How TC has effects within the cell remains unclear, but our data indicate that it affects both calcium entry and protein expression, and we suggest that it or a metabolite may be activating intracellular signaling pathways.

In summary, this study demonstrates that in ICP, UDCA may help protect the fetus by preventing the transfer of maternal bile acids to the fetus and by attenuating their vascular and other intracellular effects. This study provides mechanistic insight into the use of UDCA as a therapy in ICP, and this may allow the development of more effective interventions.

## Supplementary Material

This article includes supplemental data. Please visit *http://www.fasebj.org* to obtain this information.

## Abbreviations

^3^H-ES: ^3^H-estrone sulfate
^3^H-TC: ^3^H-taurocholic acid
CRC: concentration response curve
GO: gene ontology
ICP: intrahepatic cholestasis of pregnancy
OAT: organic anion transporter
OATP: organic anion–transporting polypeptide
PSS: physiologic salt solution
T7: bacteriophage T7 promoter
TC: taurocholate
SLCO4A1: solute carrier organic anion transporter family member 4A1
UDCA: ursodeoxycholic acid
